# Nutrition care is an integral part of patient-centred medical care: a European consensus

**DOI:** 10.1007/s12032-023-01955-5

**Published:** 2023-03-07

**Authors:** Nicole Erickson, Erin Stella Sullivan, Marianna Kalliostra, Alessandro Laviano, Joost Wesseling

**Affiliations:** 1 Comprehensive Cancer Center Munich, Ludwig Maximilian University of Munich Hospital, Munich, Germany; 2 Writing Group, The European Union Thematic Network on Integrated Nutrition in Cancer Care (INC2), Croydon, UK; 3 European Federation of the Associations of Dietitians (EFAD), Naarden, The Netherlands; 4 School of Food and Nutritional Sciences, University College Cork, Cork, Ireland; 5 The European Nutrition for Health Alliance, London, UK; 6 Department of Translational and Precision Medicine, Sapienza University of Rome, Rome, Italy

**Keywords:** Multidisciplinary team, Nutrition, Malunutrition, Cancer, Clinical outcomes, European beating cancer plan, Patient-centred care, Dietitian

## Abstract

While healthcare is becoming more patient-centred, evidence-based nutrition interventions are still not accessible to all patients with cancer. As nutrition interventions directly improve clinical and socioeconomic outcomes, patient-centred care is not complete without nutrition care. While awareness of the negative impact of malnutrition on clinical outcomes, quality of life, and functional and emotional wellbeing in cancer is growing, there is relatively poor awareness amongst patients, clinicians, policymakers, and payers that nutrition interventions -particularly those begun in the early stages of the disease course- are an effective method for improving such outcomes. The European Beating Cancer Plan recognises the need for a holistic approach to cancer but lacks actionable recommendations to implement integrated nutrition cancer care at member state level. When considering nutrition care as a human right, the impact on quality of life and functional status must be prioritized, as these may be equally as important to patients, especially in advanced cancer where improvements in clinical outcomes such as survival or tumour burden may not be attainable. We formulate actions needed at the regional and the European level to ensure integrated nutrition care for all patients with cancer. The 4 main Take Home Messages are as follows: 1. The goals of Europe’s Beating Cancer Plan cannot be achieved without integrating nutrition across the cancer care continuum. 2. Malnutrition negatively impacts clinical outcomes and has socioeconomic consequences for patients and healthcare systems. 3. Championing integrating nutrition care into cancer care is therefore the duty and ethical responsibility of clinicians (Hippocratic Oath—primum non nocere) and 4. Nutrition care is a cost effective, evidence-based therapy.

## Introduction

Patient-centred care (PCC) requires a shift in focus from the traditional disease-focused, clinician-centric model of care to a system which empowers and enables patients to participate in shared decision-making and self-management [1–4]. While healthcare professionals (HCPs) generally appreciate the value of PCC, many may overestimate the level of PCC being achieved in practice. Despite strong evidence for improvements in patient-reported and clinical outcomes, nutrition is poorly integrated into multidisciplinary care in many areas of medicine, including cancer care [5–16]. Unfortunately, throughout Europe, there is a lack of consistent, coordinated integration of nutrition care throughout the cancer care continuum, and as such, PCC implementation has not been truly achieved. Nonetheless, the concept of PCC is considered so essential in cancer care that the Institute of Medicine listed PCC as the first of six interconnected essential components for the delivery of high-quality cancer care [17]. These recommendations quickly gained traction throughout major medical institutions across the world and were integrated into their guidelines, including those for accreditation by the Organisation of European Cancer Institutes (OECI) [18–20]. Recognition of the fact that PCC is not complete, nor as effective without nutrition care is that what differentiates a good physician from a great physician [21].

Suboptimal integration of nutrition into cancer care not only overlooks the value of PCC including nutrition interventions on quality of life (QoL), but also the fact that medical care itself is less effective when the patient is nutritionally depleted. While awareness of the negative impact of malnutrition on clinical outcomes in cancer is growing, there is relatively poor awareness amongst clinicians and patients alike, about the role of nutrition in improving clinical outcomes despite an ever growing body of strong clinical evidence. [5, 22–27]. In addition to tangible positive impacts on survival, length of stay and tolerance to treatment, the QoL of people living with, and beyond cancer, is positively impacted by targeted nutrition interventions [28]. The European Beating Cancer Plan calls for a holistic approach to cancer, from prevention and early diagnosis to treatment and quality of life of patients and survivors [29]. PCC, which includes nutrition, is particularly important when patients are faced with a devastating diagnosis and/or a chronic disease such as cancer, and where *quality* of life is potentially more modifiable than *quantity* of life. The aim of this paper is therefore to highlight the clinical evidence, the ethical considerations, the economic advantages, and the patient perspectives with respect to nutrition care as an integral part of the cancer care continuum.

## Improving nutrition status directly impacts clinical outcomes

High-quality medical care requires full integration of nutrition care into all steps of the cancer care pathway [30, 31]. Consistent evidence derived from randomized controlled trials shows that integrating nutrition care into cancer care positively impacts clinically relevant outcomes including reduction of toxicities, reduced post-operative complications, increased progression free survival and overall survival [32–34]. The impact of malnutrition on mortality in cancer has long been recognised and in 1932, an autopsy study of 500 cancer patients at Harvard Medical School found cachexia to be the primary cause of death in 22% of cases, and a complicating factor in many more [35]. Given that the landscape of oncology has dramatically changed since the twentieth century, these statistics are unlikely to accurately represent the natural history of cancer-related malnutrition today, however, plausible biological mechanisms have been proposed which would suggest causality in the cachexia-death relationship [36]. Furthermore, the metabolism of many anti-cancer treatments is negatively impacted by reduced muscle mass, and the resulting side effects compromise quality of life and impair physical performance, or even shorten survival due to dose-limiting toxicities interrupting the intended treatment plan [37, 37–63]. Therefore, nutrition status is an essential consideration in ensuring optimization of the efficacy of standard medical, radiation and surgical oncology interventions.

Aspects of PCC are components of the internationally recognized quality standards for nutrition care—yet the integration of professionally delivered evidence-based nutrition care into the multidisciplinary team is lacking across the European Union and globally [5–16]. In fact, it has been more than 40 years since awareness of the importance of nutrition status in hospital settings was widely acknowledged as the ‘skeleton in the hospital closet’ [64] and a large body of supporting evidence has accumulated. Nonetheless, consistent improvement in nutrition care has not materialised [65]. Relative to astounding contemporary investment in medical oncology research, with 52% of 56,000 molecules in the current drug development pipeline being for cancer [66], there is comparatively little investment in non-pharmacological or complex behavioural interventions. Moreover, when non-pharmacological studies are conducted, they are especially prone to exclusion from meta-analyses, due to high levels of heterogeneity (in methodology, outcomes, interventions and reporting) which is confounded by a lack of consensus on the appropriate methodological approach to evaluating complex interventions [67]. As such, despite a large body of individual studies demonstrating the value of early nutrition care in cancer, the overall body of evidence appears to remain inconclusive due to the relatively low number of studies which can be included at the level of a systematic review or meta-analysis. However, despite a *relative* lack of investment and a disproportionate focus on the legitimate difficulty in reversing refractory cachexia [68], perhaps the most important point to note is that there is consistent, robust evidence demonstrating the ability of a variety of early nutrition interventions to significantly improve clinical outcomes [28, 60, 69–71]. Clinicians, patients, and stakeholders at every level need to understand that evidence-based patient-centred care includes nutrition and as such, its omission is a disservice to people living with and beyond cancer.

## Integrating nutrition into cancer care is evidence-based

The impact of nutrition status throughout the care continuum on QoL, functional and emotional well-being, and on clinical outcomes have been underappreciated in favour of quantifiable, measurable end-points of interest to pharmaceutical regulators [72–77]. Despite strong recommendations for multimodal management of cancer-related malnutrition, the implementation of such multidisciplinary care is in its infancy [7, 78–84].

A growing body of evidence reflects that nutrition status has a significant effect on clinical outcomes and QoL. A large majority of patients experience some form of nutrition-related issues during their cancer journey [6, 7, 85, 86]. Moreover, long-term side effects following curative treatment commonly include body composition changes, nutrition impact symptoms and functional limitations which, in turn, impact QoL and are amenable to rehabilitation or supportive care [87]. Physicians and other members of the MDT such as nurses should therefore regularly prescribe nutrition care to cancer patients at all stages of care, whether it be providing first-line advice, or referring to specialists such as oncology dietitians [30, 88–91].

## Integrating nutrition into cancer care is a human right

The WHO Ministerial Conference on Nutrition and Noncommunicable Diseases in the Context of Health 2020 led to the Vienna Declaration which urges the mandating of person-centred nutrition care [92] and the International Working Group for Patients’ Right to Nutritional Care (composed of experts in clinical nutrition and representatives of international nutrition organizations) argue that nutrition care is an emerging human right that lies at the intersection of the existing human right to food and the human right to health, where patients hold the ‘right to be fed’ [93]. A right-holder implies duty-bearers, in this case, the state, policymakers, institutional managers and caregivers, are ethically responsible and must be held accountable. As the performance of these professionals is of critical importance, nutrition education is a priority [94, 95]. However, this can only be achieved by developing an institutional culture that values nutrition care and recognizes the need for a multi-stakeholder approach. Ethical debates such as when to withdraw nutrition and hydration in end-of-life settings [96–98] are in fact, only applicable if we introduce nutrition care in the first place.

According to the Europe’s Beating Cancer Plan all cancer inequalities should be reduced across the EU [29]. The inalienable right to high-quality PCC care, regardless of geographic or economic region, to avoid unnecessary deaths and suffering from cancer is supported by the European Charter of Patient Rights [99]. Malnutrition is not a fringe issue, and affects large amounts of cancer patients [100, 101]. Independent of potential sequelae and impact on medical outcomes which might also be improved with nutrition [69], malnutrition has also been documented as its own psychosocial challenge [102, 103].

## Integrating nutrition into cancer care is economically advantageous

Cancer care can affect the economic circumstances of patients and their households. QoL, costs of treatment and survival can be significant and lead to further inequalities [104, 105]. Effective cancer control programs and policies should therefore consider economic aspects for all cancer patients, survivors, and their carers [106–111]. A poor nutrition status is costly [106, 112, 113] and nutrition interventions contribute to reduced healthcare costs [112, 114–117]. On the other hand, the economic cost of malnutrition also impacts the healthcare system due to the costs associated with more complications, increased length of hospital stay (LOS), readmissions and increased morbidity [112]. In the UK, it was found that the cost of treating a malnourished patient is up to 3 times higher compared to a non-malnourished patient [112] Data from the Netherlands reveal that managing disease-related malnutrition accounted for 4.9% of total healthcare expenditure [118]. US economic modelling based on international health economics data suggested that widely implementing nutrition support in gastrointestinal cancer alone could account for up to US $242 million in Medicare savings each year [119]. Importantly, these savings have been demonstrated in real-world settings, showing that significant cost savings can be achieved by implementing optimal nutrition care in cancer [114, 115, 120].

## Accessible patient-centred nutrition information empowers patients to take action and improves quality of life

Nutrition has been identified as an important and essential factor for empowering patients because it internalises their locus of control (LOC), supporting development of self-efficacy at a time where patients can experience a loss of bodily autonomy and seek self-management strategies which re-embody a sense of control. With these psychological considerations in mind, PCC demands that the right information is provided, in the right way, at the right time to the right patients. Dietitian-led nutrition care aims to achieve exactly this goal and should therefore not be overlooked [121–126].

To ensure improved outcomes and prevent recurrence, it is essential that nutrition education addresses patients’ weight management goals during and after treatment, to ensure patients do not fall prey to inappropriate, non-evidence-based nutrition advice which is widespread, easily accessible online, and frequently promoted by unqualified ‘experts’ [127–132]. Patient-centred consultations utilizing effective communication strategies are thus essential for increasing awareness about the consequences of cancer diets and encourage informed decision-making. This is especially important as patients consistently report lack of access to nutrition professionals while simultaneously reporting a lack of communication about nutrition on the part of their physicians—even when questions are directly posed [6, 133, 134].

In fact, a European survey including 907 cancer patients and survivors showed that not only is access to nutrition care lacking, but also that when patients are left alone they seek nutrition information elsewhere often finding information that is not evidence-based, possible harmful and sometimes counterproductive [85]. Even information presented on cancer centres websites does not meet the universal health literacy standards [135, 136], Patients with high unmet information needs are more likely to seek out unproven complementary and alternative medicine such as restrictive diets which may potentially negatively impact nutrition status [137, 138].

Unfortunately, psychological analyses have shown that short answers without explanations make patients feel that their needs have not been acknowledged or deemed important. This can lead to patients becoming more cemented in their original, incorrect beliefs [139–142]. On the other hand, a scientifically sound answers, and more importantly, decisive identification of alternatives encourages good decision-making by patients. Such communication strategies need to be strengthened among physicians—especially with regard to nutrition advice, as many patients will never see a dietitian, and depend on this first-line advice from the clinician with whom they have most contact [143]. Where knowledge on the part of physicians is lacking, the value of a specialised dietitian as part of the multidisciplinary team becomes even more apparent. It is therefore essential that oncologists and members of the multidisciplinary care teams understand their own scope of practice with respect to medical nutrition therapy, and appropriate referral procedures are standard practice [11, 22, 144–148]. In this context, nurses are often the most accessible member of the MDT to patients. They have an essential role in ensuring access to nutrition care, in particular for non-complex cases where they can provide invaluable first-line advice, or in complex cases, where they are responsible for implementing dietitian delivered recommendations. This example demonstrates why all members of the MDT must be able and willing to champion nutrition care, within the scope of their own profession. Moreover, it highlights why nutrition education and training cannot be limited to the nutrition specialist, if our aim is for widely accessible, properly implemented, integrated nutrition care in cancer.

Importantly, patients must be made aware of the importance of good nutrition in cancer and that intervention can positively impact their personal and clinical outcomes. Without this knowledge, patients are not able to advocate for themselves and identify red-flag signs. Additionally, their cancer team should encourage and empower patients to engage in shared decision-making and be confident to request assessment and treatment of nutrition-related concerns as they arise [149, 150].

Eating is a social process and contributes to quality of life [69, 102, 121, 151–155]. Optimal nutrition care is a human right and cannot be delivered in the absence of effective communication strategies [156, 157].

## Patient-centred care means listening to patient voices

**Fig. 1:**
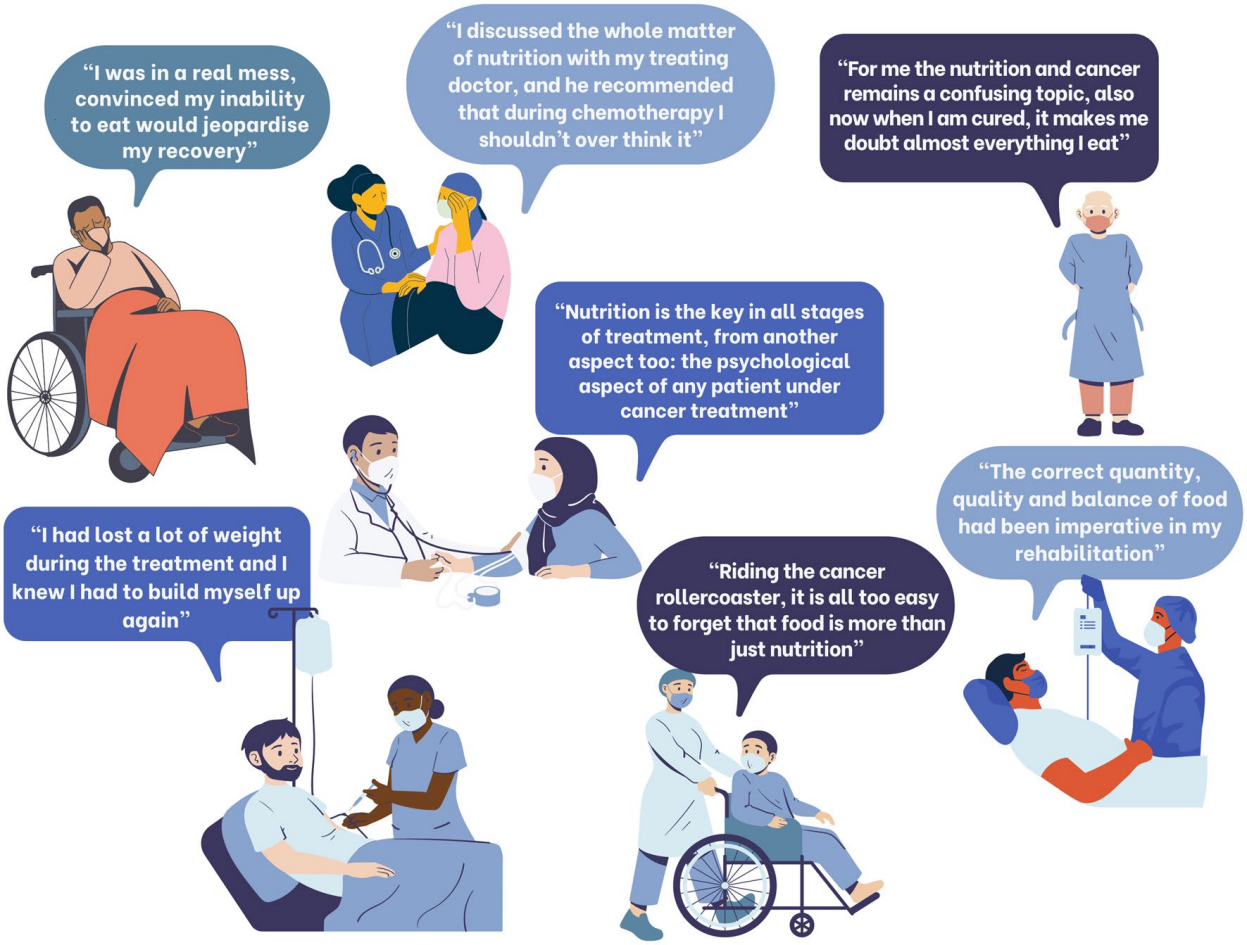
Patient quotes (adapted with permission from [160])

**Fig. 2:**
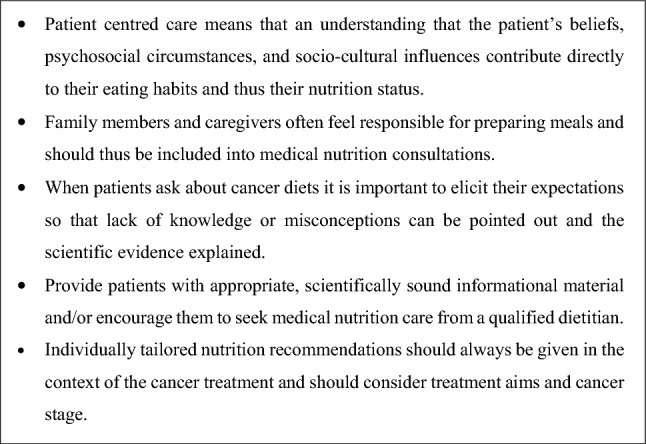
Info box for patient-centred communication regarding nutrition and cancer

In order to practice patient-centred care, it is essential to listen to and to understand what is important to patients. Many studies have shown that patients are not getting the nutrition care they need, and desire [6, 22, 85, 133, 158]. The European Cancer Patient Coalition (ECPC) recently presented rich qualitative data collected via direct patient interviews which indicate the central role of good nutrition care in cancer, from the patient perspective [159, 160]. Figure 1 includes a selection of quotes from the ECPC booklet showing patients’ points of view in their own words [160]. The patients’ insights highlight the fact that patients recognise the need for nutrition care, but are unfortunately, not consistently receiving the personalised nutrition counselling required to implement meaningful changes in their daily life. The Information Box depicted in Fig. 2 provides guidance to patient-centred communication with regards to nutrition and cancer.

## Integrating nutrition into cancer care requires a plan

Scaled integration of these and other emerging nutrition interventions into healthcare would require significant economic investment and continued rigorous research but in the end the cost benefit is proven[17, 114]. To implement integrated nutrition care in cancer, coordination of messaging is needed. To support policy makers, service providers, and patient advocates the following first steps are recommended:

- **Step 1** Widespread advocacy is required to raise awareness amongst clinicians and patients. It should be communicated that nutrition care in cancer has a significant impact on both clinically relevant outcomes and health-related quality of life issues which are central to PCC. This message needs to reach clinicians whose referral practices to dietetics require changes. Patients need to understand this message so they can then self-advocate when they identify that nutrition has become a problem for them. Finally, this message needs to be communicated to policymakers or healthcare management whose buy-in is needed to acquire funding and coordinate human and infrastructural resources to facilitate development and ongoing provision of comprehensive dietetic services in oncology. This call to action should refer to the key aspects of evidence, human rights and economic value outlined above.
- **Step 2** Cancer services should urgently incorporate key performance indicators (KPIs) into their regular quality assurance systems to benchmark and audit adherence to evidence-based nutrition recommendations. These KPIs should be evidence-based or at least based on expert consensus. ESPEN, ESMO, COSA and others have many such guidelines [30, 81, 88, 89, 161] which could be used as starting points to develop a quality assurance standards for Integrated Nutrition Care in Cancer. At an absolute minimum, audits should report on malnutrition screening, oncology specific dietetic staffing and availability of nutrition assessment for all patients with high-risk diagnoses (e.g., head & neck cancers, gastrointestinal cancers, high-dose chemotherapy, radiotherapy to the head & neck or pelvis).
- **Step 3** At the European level, several of the actions of the Beating Cancer Plan should include nutrition, notably, the plan mentions the role of diet and exercise in cancer prevention but does not focus on the specific role of nutrition within the management of cancer. In order to maximise the effect of a number of the flagship initiatives arising from the Beating Cancer Plan [29], ‘National Comprehensive Cancer Centre’ accreditation should be associated with a minimum acceptable level of nutrition and dietetic service provision, in which all cancer patients are nutritionally screened, the dietitian is a core part of the multidisciplinary team, and all members of the MDT receive basic and regular nutrition training. The ‘Knowledge Centre on Cancer’, ‘Inter-Specialty Training Programme’, ‘Cancer Diagnostic and Treatment for All’, ‘Partnership on Personalised Medicine’, ‘Better life for cancer patients’, ‘Cancer Inequalities Registry’, “EU-Network of Comprehensive Cancer Centres” and ‘Guidelines and Quality Assurance’ initiatives must explicitly include nutrition care.

## Conclusion

Across the cancer continuum, and especially for people living with incurable disease, the improvement of QoL may be more significant to patients than improvement in traditionally ‘clinically relevant’ outcomes such as tumour burden or overall survival [75]. Thus, when considering nutrition care as a human right, it is only appropriate to think about the outcomes which matter to patients themselves and the impact of malnutrition on QoL and how nutrition and nutrition-related issues affect cancer patients in general.

The pivotal role of nutrition care particularly applies to cancer care and is founded on the basis that anti-cancer treatments can be more effective in patients with a balanced nutrition status, leading to less delays in treatment, and less dose-limiting toxicities [42, 162]. However, across the cancer care continuum, and for the increasing group of people living with and beyond cancer, as well as those who live with the chronic ‘late effects’ of cancer appropriate and timely nutrition care still remains a documented unmet need [163–169].

Nutrition care in cancer is an essential component of standard cancer care and is a basic right for people living with and beyond cancer. Therefore, these initial recommendations on advocacy, evidence, quality assurance and European actions should function as an essential guide to ensuring that Europe’s Beating Cancer Plan and other health programmes and policies have nutrition care at its centre.

According to Europe’s Beating Cancer Plan, adopted in 2021, the European Commission aims to reduce all cancer inequalities across the EU [29]. Equal access of all cancer patients to high-quality care, regardless of geographic or economic region, to avoid unnecessary deaths and suffering from cancer, is further supported by the European Charter of Patient Rights [99]. However, professionals should not only provide mere access to high-quality care, including nutrition, but take responsibility to *educate* patients and *involve* them in decision-making to ensure their individual needs are met.

## Data Availability

No data were generated in the production of this manuscript.
